# Effects of Statin Use in Advanced Chronic Kidney Disease Patients

**DOI:** 10.3390/jcm7090285

**Published:** 2018-09-17

**Authors:** Tao-Min Huang, Vin-Cent Wu, Yu-Feng Lin, Jian-Jhong Wang, Chih-Chung Shiao, Likwang Chen, Shih-Chieh Jeff Chueh, Eric Chueh, Shao-Yu Yang, Tai-Shuan Lai, Shuei-Liong Lin, Tzong-Shinn Chu, Kwan-Dun Wu, On Behalf of the National Taiwan University Hospital Study Group on Acute Renal Failure (NSARF)

**Affiliations:** 1 Division of Nephrology, Department of Internal Medicine, National Taiwan University Hospital, Zhongzheng, Taipei 100, Taiwan; taomin.huang@gmail.com (T.-M.H.); q91421028@ntu.edu.tw (V.-C.W.); dr.yufenglin@gmail.com (Y.-F.L.); yangshaoyo@yahoo.com.tw (S.-Y.Y.); taishuanster@gmail.com (T.-S.L.); linsl@ntu.edu.tw (S.-L.L.); tschu@ntu.edu.tw (T.-S.C.); kdwu@ntuh.gov.tw (K.-D.W.); 2 Graduate Institute of Clinical Medicine, National Taiwan University College of Medicine, Zhongzheng, Taipei 100, Taiwan; 3 Division of Hospital Medicine, Department of Internal Medicine, National Taiwan University Hospital, Zhongzheng, Taipei 100, Taiwan; 4 Division of Nephrology, Department of Internal Medicine, Chi Mei Medical Center, Liouying, Tainan 736, Taiwan; win7a@yahoo.com.tw; 5 Division of Nephrology, Department of Internal Medicine, Saint Mary’s Hospital, Loudong, Yilan 265, Taiwan; chungyy2001@yahoo.com.tw; 6 Institute of Population Health Sciences, National Health Research Institutes, Zhunan, Miaoli 350, Taiwan; 7 Cleveland Clinic Lerner College of Medicine and Glickman Urological and Kidney Institute, Cleveland Clinic, Cleveland, OH 44106, USA; jeffchueh@gmail.com; 8 Case Western Reserve University, No. 10900 Euclid Ave., Cleveland, OH 44106, USA; ericc0109@gmail.com

**Keywords:** statin, sepsis, chronic kidney disease, diabetes, major adverse cardiovascular events, mortality

## Abstract

Although statin treatment is recommended for patients with chronic kidney disease (CKD) stages I–IV, its potential benefits have not been reported in advanced CKD patients. Non-diabetic patients with advanced CKD (pre-dialysis patients, estimated glomerular filtration rate <15 mL/min/1.73 m^2^) were enrolled from a National Health Insurance Research Database with a population of 23 million. Statin users and non-users were matched using propensity scoring and analyzed using Cox proportional hazards models, taking mortality as a competing risk with subsequent end-stage renal disease (ESRD) and statin doses as time-dependent variables. A total of 2551 statin users and 7653 matched statin non-users were identified from a total 14,452 patients with advanced CKD. Taking mortality as a competing risk, statin use did not increase the risk of new-onset diabetes mellitus (NODM) or decrease the risk of de novo major adverse cardiovascular events (MACE), but reduced all-cause mortality (hazard ratio (HR) = 0.59 [95% CI 0.42–0.84], *p* = 0.004) and sepsis-related mortality (HR = 0.53 [95% CI 0.32–0.87], *p* = 0.012). For advanced CKD patients, statin was neither associated with increased risks of developing NODM, nor with decreased risk of de novo MACE occurrence, but with a reduced risk of all-cause mortality, mainly septic deaths.

## 1. Introduction

The burden of chronic kidney disease (CKD) is estimated to affect 26 million people in the USA and continues to increase globally [1]. A significant amount of CKD and end-stage renal disease (ESRD) patients on dialysis succumb to accelerated cardiovascular disease (CVD) [2]. Meta-analyses and observational studies have disclosed a 1.4–3.7-fold increase in mortality from CVD among patients with CKD compared to those without CKD [2].

Recently, meta-analysis studies have shown that statin (3-hydroxy-3-methylglutaryl coenzyme A [HMG-CoA]) reductase inhibitors therapy prevents major adverse cardiovascular events (MACE) in patients with CKD stages I-IV [3], but there was little or no benefit for MACE prevention in patients undergoing dialysis [4,5]. As such, according to the Kidney Disease Improving Global Outcomes (KDIGO) CKD guideline, dialysis-dependent patients should not initiate statin therapy [6]. However, whether or not the use of statins is effective against MACE and all-cause mortality among advanced CKD (pre-dialysis patients, estimated glomerular filtration rate <15 mL/min/1.73 m^2^) patients is still unknown. The the long-term use of statins is associated with an increase in hyperglycemia and overt diabetes mellitus [7]; additionally, studies that have assessed the benefits of statins have not extensively discussed the risk of new-onset diabetes mellitus (NODM) for patients with advanced CKD [8]. Furthermore, exposure to statins may have a protective effect against the development of sepsis and decrease mortality in critically ill patients [9], and yet such results have not been concluded in advanced CKD patients.

Thus, we aimed to evaluate the effects of statin use on NODM, de novo MACE, or all-cause mortality including cardiovascular-related deaths and severe sepsis-related deaths among advanced CKD population in a nationwide cohort.

## 2. Materials and Methods

### 2.1. Data Source

This population-based cohort study retrieved data from the Taiwan National Health Insurance (TNHI) Research Database, which is one of the largest health insurance databases in the world and has been used extensively in various epidemiologic studies [10,11]. The TNHI covers almost all 23.7 million people in Taiwan and contains comprehensive healthcare information regarding outpatient visits, hospital admissions, disease profiles, prescriptions, interventional procedures, and vital status. All diagnoses are based on the codes of the International Classification of Diseases, 9th Revision (ICD-9).

### 2.2. Study Population

**Figure 1:**
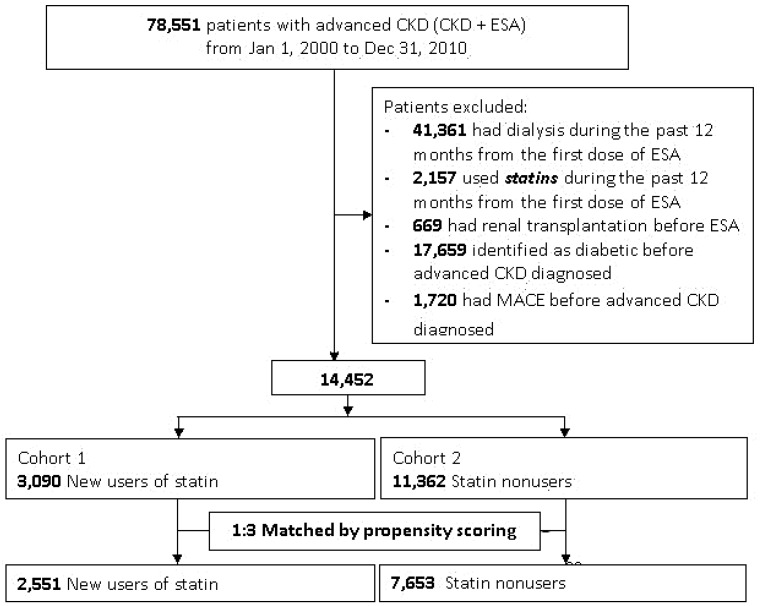
Detailed flowchart for patient enrollment. Abbreviations: CKD, chronic kidney disease; ESA, erythropoiesis-stimulating agents; MACE, major adverse cardiac events.

This study retrospectively enrolled patients aged ≥20 years who had diagnosis codes for CKD and were treated with erythropoiesis-stimulating agents (ESAs) from 1 January 2000 to 31 December 2010. According to the TNHI reimbursement policy, ESAs can only be prescribed in predialysis CKD patients with anemia who have a hematocrit level of ≤28% and a serum creatinine level of > 6 mg/dL (equivalent to an estimated glomerular filtration rate (eGFR) of <15 mL/min/1.73 m^2^, CKD stage 5) with a goal of maintaining a hematocrit level of 33%–36%. We defined this combination of CKD diagnosis codes and predialysis patients using ESAs as ‘advanced CKD’. We only included advanced CKD patients who were new users of statins after the first prescription of ESAs. Patients who had been treated with dialysis (identified by procedure codes) or underwent renal transplantation before ESA prescription and those who did not survive 90 days after the first ESA therapy were excluded (Figure 1).

Patients with NODM (no pre-existing diabetes mellitus at enrollment) during the follow-up period were identified. To observe the effect of statins on the prevention of cardiovascular events in these advanced CKD patients, we also excluded patients who had MACE recorded before advanced CKD enrollment. As such, this also prevented ICD codes of prior MACE to carry over, in order to avoid misclassification bias. Each drug dispensing is registered based on international classification of drugs, the Anatomical Therapeutic Chemical (ATC) system, as well as the date of dispensing, quantity dispensed, strength, and formulation. The baseline comorbidities, including diabetes and CKD, were identified from at least one in-patient claim or three ambulatory visits within one year preceding the first ESA treatment. This identification approach has been validated with good predictive power [10,11,12]. The Charlson comorbidity index was also calculated by weighing baseline comorbidities [13].

### 2.3. Outcome Measures

The primary outcome was all-cause mortality, MACE-related or severe sepsis-related deaths. Sepsis was defined according to the American College of Chest Physicians/Society of Critical Care Medicine (ACCP/SCCM) as a systemic inflammatory syndrome in response to infection, which when associated with acute organ dysfunction is said to be severe [14] (Supplementary methods). The secondary outcome was NODM and de novo MACE after statin use. NODM was defined vianew prescriptions for any anti-diabetic drugs (oral agents or insulin) together with the ICD code of diabetes while the outcome of diabetes was defined by any prescriptions for diabetes drugs (oral agents or insulin) in consecutive visits (>30 days apart) [15,16,17]. De novo MACE included non-fatal myocardial infarction (MI), coronary artery bypass graft (CABG), stroke and coronary angiography [18]. All patients were followed from the date of the first ESA prescription to the date of the first occurrence of the outcome event, and censored at either death, transplantation or the end of the study (31 December 2011), whichever occurred first.

### 2.4. Exposure Assessment

We identified statin treatment in accordance with the prescription for statins during the study period, and restricted our analysis to new users by requiring a period of at least one year without any prescription for statins before the diagnosis of advanced CKD. The statin users and non-users were matched at a 1:3 ratio by propensity scoring, in an attempt to make an unbiased estimate of all the baseline confounders (Figure 1). The doses of statin were converted to the number of defined daily doses (DDDs) as defined by the World Health Organization [19] using the time-span of 60 days preceding outcomes. A DDD is equal to atorvastatin 20 mg (supplementary methods).

### 2.5. Ethics Committee Approval

As all personal information is de-identified in the TNHI Research Database, informed consent was waived and this study was exempt from a full ethical review by the institutional review board of the National Taiwan University Hospital (201212021RINC).

### 2.6. Statistical Analysis

Continuous variables were presented as the mean ± standard deviation (SD) or median, as appropriate. We performed a propensity score matching (PSM) to reduce the selection bias due to the baseline differences between the statin users and non-users. First, we conducted multivariable logistic regression analysis to identify the predictors for the prescription of statin. Baseline comorbid conditions, age, and sex (listed in Table 1) were inputted into a non-parsimonious multivariable logistic regression model to predict the prescription of statin. The predicted probability derived from the estimated equation was the propensity score for each individual (Table S1). Then, we used the “Match ()” function in the “Matching” library of the R statistical software to do PSM based on the default value of “caliper” (i.e., caliper = 0.25) and the Mahalanobis distance without replacement. After the matching, the standardized mean difference (SMD) of all variables were <0.1 (Table 1).

We evaluated the risk factors of outcomes and death using Cox proportional hazards models with all statins and ESRD as time-dependent covariates to account for their impact. Time-dependent analytical methods have been shown to avoid time bias in observational cohort studies [18]. Variable selection for Cox regression hazards modeling was performed using step-wise multiple regression, with a *p*-to-enter and *p*-to-leave both equal to 0.15. The validity of the proportional hazard assumption, linearity of continuous variables, and lack of interaction were found to be valid unless otherwise indicated. Because of the higher mortality rate and metabolic disease in CKD patients, competing-risk regression using the Fine and Gray model by considering the subdistribution hazard was also performed.

After adjustment for comorbidities, we also conducted an adjusted comparison of risks for statin-associated NODM or mortality among patient subgroups stratified by the status of comorbidities, subsequent ESRD, and category of statins, as shown in the forest plot.

To evaluate the effect of statin dose on the risk of all-cause mortality, we further adopted a generalized additive model (GAM) with adjustments for baseline comorbidities and the risk of mortality. This method grants adjustments for possible non-linear effects from continuous variables [6]. The result was shown as a function curve with values of the log odds ratio and was centered to have an average of zero over the range of the data.

All analyses were carried out using R software, version 3.1.2 (Free Software Foundation, Inc., Boston, MA, USA). A two-sided *p*-value < 0.05 was considered significant.

## 3. Results

### 3.1. Patient Characteristics

Among advanced CKD patients before PSM, statin users (*n* = 3090) were more frequently females, younger, and had lower rates of comorbidities (except hypertension) than statin non-users (*n* = 11,362). After PSM (1:3 ratio, Figure 1), 2551 statin users were compared with 7653 statin non-users as controls. The average ages, gender distributions and Charlson comorbidity indeices were not significantly different, and the proportions of comorbidities, antihypertensive drugs, and major medication uses were similar between the two groups (Table 1).

### 3.2. Effect of Statins on NODM

After a mean follow-up of 5.3 ± 3.1 years, the unadjusted rate of NODM was not significantly different between statin users and non-users. For multivariate time-dependent Cox regression analysis, the use of statin increased the risk of NODM before (HR = 1.45, 95% CI, 1.16–1.82, *p* = 0.001) and after PSM (HR = 1.46, 95% CI, 1.14–1.85, *p* = 0.002) compared to statin non-users. However, when taking the competing risk for mortality into account for the time-dependent model analysis, statin use did not augment the risk of NODM before or after PSM (Table 2).

### 3.3. Effect of Statins on De Novo MACE

For de novo MACE, statin usage was not protective in comparison to non-users in univariate analysis. Furthermore, statin users among advanced CKD patients did not have a reduced risk of de novo MACE both in the adjusted and competing risk models (Table 2). In regard to de novo MACE, advanced CKD patients either with (HR = 1.21, 95% CI, 0.8–1.84, *p* = 0.645) or without (HR = 1.25, 95% CI, 0.7–1.95, *p* = 0.701) subsequent dialysis also could not benefit from statin treatment.

### 3.4. Effect of Statins on Mortality

After PSM, the risk of all-cause mortality was improved following statin use among advanced CKD patients (HR = 0.59, 95% CI, 0.42–0.84, *p* = 0.004). On identifying the etiologies attributed to mortality, statin users had reduced odds of sepsis-related mortality (HR = 0.53, 95% CI, 0.32–0.87, *p* = 0.012), although MACE-related mortality was not significantly reduced (HR = 1.75, 95% CI, 0.87–3.13, *p* = 0.065) (Table 2).

**Figure 2:**
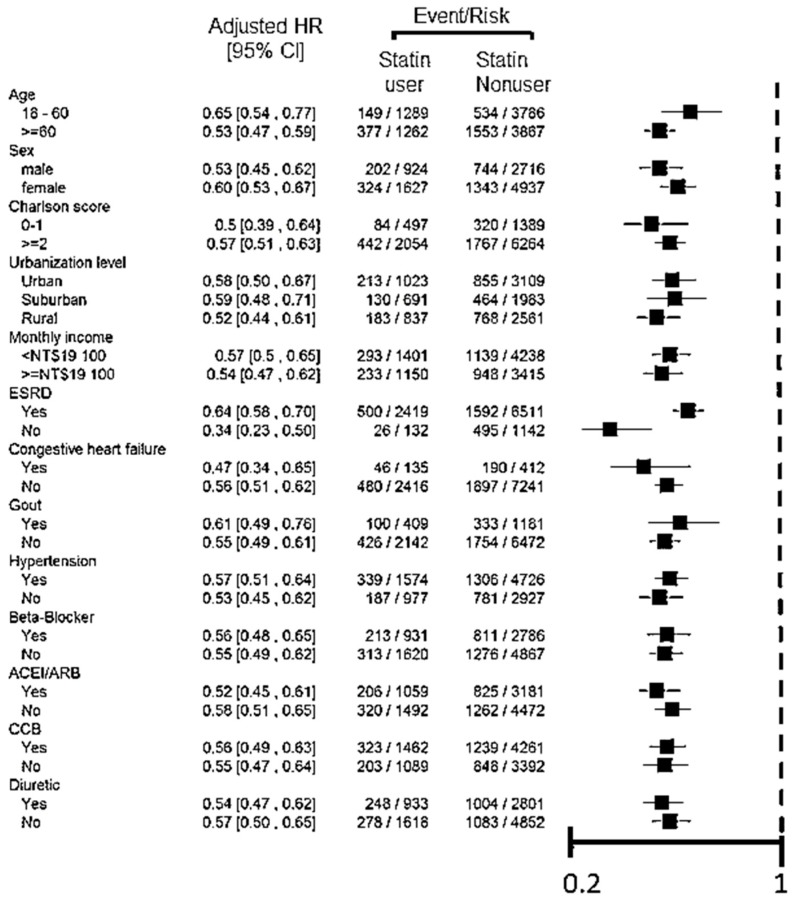
The adjusted hazard ratio for statin use versus nonuse in advanced chronic kidney disease patients on the incidence of all-cause mortality after propensity score matching, stratified by comorbidities. (ESRD, end-stage renal disease denoted who received subsequent chronic dialysis). Abbreviations: HR, harzard ratio; ACEI, angiotensin-converting-enzyme inhibitors; ARB, Angiotensin II receptor blockers; CCB, calcium-channel blocker.

From the forest plot, statin use decreased all-cause mortality in subgroups of different baseline characteristics and comorbidities consistently after PSM (Figure 2). This protective effect was significant among patients who subsequently developed a dialysis-dependent status as well as those who were pre-dialysis advanced CKD patients.

**Figure 3:**
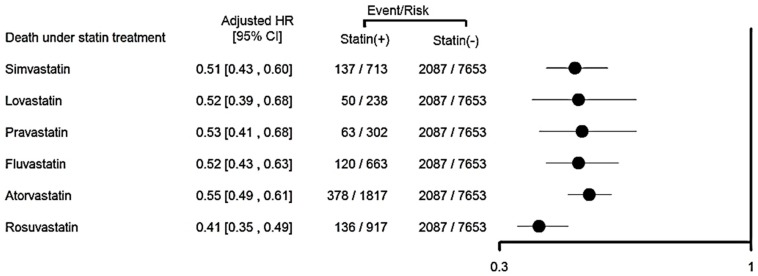
The adjusted hazard ratio for statin users versus non-users in advanced chronic kidney disease patients on the incidence of all-cause mortality with post-propensity score matching, stratified according to individual statin therapy. HR, harzard ratio.

We further assessed the risk of all-cause mortality for the class effect of various statins by forest plot analysis after PSM, and found that all statins reduced mortality for advanced CKD patients (Figure 3).

**Figure 4:**
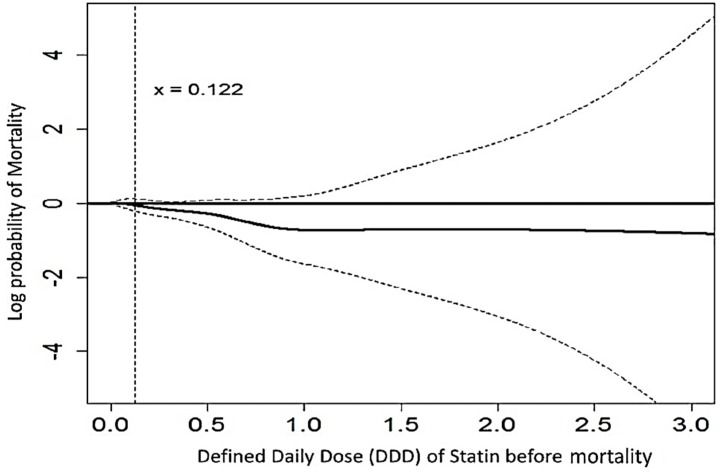
The dose-response relationship between statins and the logit probability of all-cause mortality using generalized additive modeling (GAM). The adjustments for factors are listed in Table 1. The dotted line indicates 95% confidence intervals.

Additionally, we evaluated the cumulative doses between DDD before the event and the risk of all-cause mortality utilizing a GAM analysis. The function curve was nonlinear and there was a significant trend approaching decreased all-cause mortality, especially for a daily statin dose >0.122 DDD (equal to 2.4 mg atorvastatin) within a time-span of 60 days, before leveling out after approximately 1.0 DDD (Figure 4).

## 4. Discussion

Our study supports the clinical benefit of statin therapy among advanced CKD patients in the reduction of all-cause mortality and especially sepsis-related mortality. In addition, after the competing risk for mortality taken into account, statin therapy was not associated with an increased risk of NODM, nor was it associated with a decreased incidence of de novo MACE.

Although statin use is associated with lower incidence of CVD in the general population or even in patients with mild to moderate CKD [2,3,20,21], our study demonstrated that the primary prevention by statins of lowering the CVD/MACE risk among advanced CKD patients was not prominent in this analysis of a large “real-world” contemporary population-based cohort. To our knowledge, we are the first to identify the effect of new statin therapy in alleviating all-cause mortality, mainly from sepsis-related deaths for advanced CKD patients. We also present the incidence of de novo MACE and NODM after statin use in this population.

### 4.1. Statin Decreased Sepsis and Mortality

In this study, we found that statin treatment in advanced CKD patients, even those progressing to ESRD, was associated with a reduced risk of all-cause mortality, mainly from sepsis-related deaths. Statin therapy could aid the treatment and prevention of sepsis by targeting a number of inflammatory and immune-modulating cascades involved in sepsis [22]. Additionally, statins were strongly and independently associated with a reduction in the risk of sepsis events in patients who had chronic kidney disease [23]. Furthermore, statins have demonstrated the ability to reduce a number of pro-inflammatory cytokines known to be detrimental in the development and progression of sepsis and limit the coagulation response, promoting fibrinolysis in the setting of sepsis [24]. Given the current uncertainty in the medical literature about the benefits of lowering lipid levels in advanced CKD patients for reducing the incidence of mortality, our findings are timely and reassuring. We further reinforce the notion that statin treatment in a very high-risk advanced CKD population could be beneficial in reducing sepsis-related mortality. The anti-inflammatory effects of statins are associated with higher dose regents [25]; likewise, from DDD analysis in our CKD patients, the survival benefits of statins also seemed to be dose-dependent, and the reduction in risk was constant after a 1.0 DDD. Our findings may shed light on some heterogeneity of findings in previous similar studies, as all statins in this class showed a consistent protective effect against all-cause mortality.

### 4.2. NODM after Statin Treatment

While statin therapy in mild CKD patients could induce a dose-dependent risk of NODM, no such risk was evident among advanced CKD patients. Although the underlying mechanisms for the association between insulin resistance and CKD remain unclear, numerous mechanisms such as glucose dysregulation, uremic toxin retention, inflammation, abnormal mineral metabolism, hypertension, adiposity, and uremic acidosis have been implicated [26]. Potential contributors to this so-called “burnt-out diabetes” include decreased renal and hepatic insulin clearance, a decline in renal gluconeogenesis, deficient catecholamine release, diminished food intake (because of anorexia or diabetic gastroparesis) and protein-energy wasting (with resultant loss of weight and body fat) [27]. All the factors delineate to less hyperglycemia in advanced CKD patients.

### 4.3. De Novo MACE after Statin Treatment

Several reviews of statin therapy have generally concluded that lipid-lowering is beneficial in early-stage CKD, but results in ESRD differ [5,28,29]. Randomized studies have indicated that statins have a limited role in the primary prevention of CVD or mortality in patients undergoing dialysis [6]. Our study revealed that among patients with advanced CKD, there was a benefit of decreased all-cause mortality after statin use; however, the benefit on CVD prevention was limited.

Although several different medications could lower LDL-C, only regimens including statins have been convincingly shown to reduce the risk of adverse cardiovascular events in mild to moderate CKD populations [2]. However, similar observations were not found in our patients with advanced CKD. The most likely explanation of the findings from the aforementioned trials is that arterial lesions in advanced CKD patients (which also have different pathogenesis to arterial lesions in patients without CKD or mild CKD [30]) are so severe that they are unlikely to be meaningfully reduced by statins. Another possibility could be the short duration of statin treatment among our advanced CKD enrollees before their subsequent dialysis.

### 4.4. Strength and Limitations

There were limitations in the present study. First, personal information that might affect the risk of NODM, including glucose levels, smoking and alcohol habits, physical activity and body mass index, were unavailable from the TNHI Research Database. Some uncorrected confounders were not available in the database, which might potentially influence the outcome. Second, we only studied patients without pre-existing diabetes to observe the long-term effect of statins on NODM, CVD and all-cause mortality, suggesting that treatment with statins in the primary prevention of CVD, including for diabetic patients, should be investigated further. Third, our target population of patients with advanced CKD was identified by ICD-9 codes for CKD plus drug codes for ESA [31]. Given that the rate of ESA prescription was 85% in 2012 in predialysis stage 5 CKD patients according to an internal report of the Taiwan Department of Health, we believe that the patients on ESA therapy were representative and constituted the majority of stage 5 CKD patients. Fourth, since the nutritional status affects infection and mortality, we do not have the data of body mass index and serum albumin level.

Finally, the use of statins was not randomly assigned, and the factors attributed to their prescription could not be adequately assessed and accounted. However, because the NHI database contains prescription information, we were able to determine how often prescriptions were refilled as a marker of whether statins were being taken as prescribed. With PSM, patients were matched on a single propensity score representing the probability of receiving the exposure of interest given the observed baseline characteristics; this method is especially useful when treatment cohorts are dissimilar.

In this study, we used the large-scale population-based cohort database, which offered insights into the effects of statin use among advanced CKD patients that were not examined and could not be offered via the smaller-scale multicenter studies. Further randomized prospective studies are needed to confirm our results.

## 5. Conclusions

In conclusion, this large population-based cohort study provides evidence for decreased mortality among advanced CKD patients initiating statin treatment. Our analysis showed that statin use did not significantly increase the risk of NODM, nor was statin associated with decreased risk of de novo MACE among advanced CKD patients, when taking mortality into competing account. Statin could reduce the risk of all-cause mortality, mainly sepsis-related deaths. All of these findings need to be validated further with larger prospective studies focusing on advanced CKD patients.

## Figures and Tables

**Table 1:** Clinical characteristics of statin users and non-users before and after propensity score match.

	Before PSM			After PSM		
	Statin Users (*n* = 3090)	Nonusers (*n* = 11362)	SMD	Statin Users (*n* = 2551)	Nonusers (*n* = 7653)	SMD
Age	57.61 ± 13.47	63.89 ± 15.06	0.439	59.41 ± 13.2	59.73 ± 14.99	0.023
**Gender**, *n* (%)						
Women	2109 (68.25%)	5984 (52.67%)	−0.323	1627 (63.78%)	4937 (64.51%)	0.015
Men	981 (31.75%)	5378 (47.33%)		924 (36.22%)	2716 (35.49%)	
Comorbidity				
Charlson score	1.99 ± 1.12	2.15 ± 1.28	0.136	2 ± 1.15	2.02 ± 1.14	0.020
Congestive heart failure	149 (4.82%)	737 (6.49%)	−0.072	135 (5.29%)	412 (5.38%)	−0.004
Peripheral vascular disease	12 (0.39%)	82 (0.72%)	−0.045	11 (0.43%)	47 (0.61%)	−0.025
Dementia	8 (0.26%)	115 (1.01%)	−0.095	8 (0.31%)	20 (0.26%)	0.010
COPD	188 (6.08%)	889 (7.82%)	−0.068	177 (6.94%)	465 (6.08%)	0.035
Rheumatologic disease	81 (2.62%)	233 (2.05%)	0.038	53 (2.08%)	200 (2.61%)	−0.035
Peptic Ulcer	359 (11.62%)	1696 (14.93%)	−0.098	307 (12.03%)	928 (12.13%)	−0.003
Hemiplegia	2 (0.06%)	9 (0.08%)	−0.005	2 (0.08%)	6 (0.08%)	0.000
Moderate or Severe liver disease	99 (3.20%)	685 (6.03%)	−0.135	95 (3.72%)	290 (3.79%)	−0.003
Tumor	115 (3.72%)	701 (6.17%)	−0.113	108 (4.23%)	320 (4.18%)	0.003
Hypertension	1935 (62.62%)	6868 (60.45%)	0.045	1574 (61.70%)	4726 (61.75%)	−0.001
Gout	479 (15.50%)	1967 (17.31%)	−0.049	409 (16.03%)	1181 (15.43%)	0.017
Medication for hypertension				
Alpha-Blocker	298 (9.64%)	1238 (10.90%)	−0.041	248 (9.72%)	696 (9.09%)	0.021
Beta-Blocker	1169 (37.83%)	3905 (34.37%)	0.072	931 (36.50%)	2786 (36.40%)	0.002
Calcium-Channel Blocker	1765 (57.12%)	6373 (56.09%)	0.021	1462 (57.31%)	4261 (55.68%)	0.013
Diuretic	1076 (34.82%)	4740 (41.72%)	−0.142	933 (36.57%)	2801 (36.60%)	−0.001
ACEI or ARB	1332 (43.11%)	4468 (39.32%)	0.077	1059 (41.51%)	3181 (41.57%)	−0.001
Other concomitant medication						
Aspirin	99 (3.20%)	381 (3.35%)	−0.008	86 (3.37%)	229 (2.99%)	0.022
Clopidogrel	34 (1.10%)	139 (1.22%)	−0.011	32 (1.25%)	60 (0.78%)	0.027
Ticlopidine	26 (0.84%)	70 (0.62%)	0.026	13 (0.51%)	48 (0.63%)	−0.016
Dipyridamole	985 (31.88%)	3289 (28.95%)	0.064	788 (30.89%)	2368 (30.94%)	−0.001
Nitrate	8 (0.26%)	63 (0.55%)	−0.046	8 (0.31%)	35 (0.46%)	−0.023
H2 blocker	383 (12.39%)	1608 (14.15%)	−0.052	329 (12.90%)	969(12.66%)	0.007
PPI	171 (5.53%)	1054 (9.28%)	−0.143	160 (6.27%)	466 (6.09%)	0.008
Pentoxifylline	376 (12.17%)	1386 (12.20%)	−0.001	297 (11.64%)	932 (12.18%)	−0.017
Sodium bicarbonate	17 (0.55%)	43 (0.38%)	0.025	14 (0.55%)	29 (0.38%)	0.025

Values are mean ± SD or *n* (%). Abbreviations: ACEI, angiotensin-converting-enzyme inhibitors; ARB, Angiotensin II receptor blockers; COPD, chronic obstructive pulmonary disease; H2 blocker, Histamine 2 blockers; PSM, propensity score match; SD, standard deviation; SMD, standardized mean difference; PPI, proton-pump inhibitor.

**Table 2:** Risks for NODM, de novo MACE, all-cause mortality, MACE- and sepsis-related death before and after propensity score match.

		Statin Nonusers			Statin Users		Crude		Adjusted *		Competing **	
	Events	Person-Years	IR	Events	Person-Years	IR	HR (95% CI)	*p*	HR (95% CI)	*p*	sHR (95% CI)	*p*
	before PSM						users vs. non-users					
NODM	901	17,186.0	52.4	2660	46,591.5	57.1	1.21 [0.95,1.54]	0.130	1.45 [1.16,1.82]	0.001	1.16 [0.95,1.41]	0.152
*De novo* MACE	939	17,069.7	55.0	2307	46,052.7	50.1	0.94 [0.72,1.23]	0.658	1.21 [0.94,1.54]	0.137	1.11 [0.91, 1.36]	0.323
Mortality												
All-cause mortality	586	20,168.9	29.1	3772	52,220.1	72.2	0.30 [0.21,0.43]	<0.001	0.59 [0.42,0.82]	0.002	NA	NA
MACE-related death	64	20,168.9	3.2	224	52,220.1	4.3	1.77 [0.98,3.19]	0.059	1.84 [0.82,3.31]	0.073	NA	NA
Sepsis-related death	361	20,168.9	17.9	1964	52,220.1	37.6	0.29 [0.18,0.47]	<0.001	0.58 [0.37,0.91]	0.017	NA	NA
	after PSM						users vs. non-users					
NODM	773	13,851.4	55.8	1654	34,125.3	48.5	1.42 [1.11,1.81]	0.005	1.46 [1.14,1.85]	0.002	1.16 [0.94,1.45]	0.170
*De novo* MACE	829	13,661.1	60.7	1459	33,983.3	42.9	1.16 [0.89,1.51]	0.265	1.23 [0.95,1.59]	0.124	1.14 [0.93,1.4]	0.220
Mortality												
All-cause mortality	526	16,370.3	32.1	2087	38,072.3	54.8	0.55 [0.39,0.79]	0.001	0.59 [0.42,0.84]	0.004	NA	NA
MACE-related death	56	16,370.3	3.4	130	38,072.3	3.4	1.38 [0.71,2.66]	0.339	1.75 [0.87,3.13]	0.065	NA	NA
Sepsis-related death	321	16,370.3	19.6	1071	38,072.3	28.1	0.49 [0.3,0.81]	0.005	0.53 [0.32,0.87]	0.012	NA	NA

* step-wise all variables in Table 1, statin as time-varying risks. ** adjusted with age, sex, propensity score, taking mortality as the competing risk. Abbreviations: CI, confidence interval; HR, hazard ratio; sHR, subdistribution hazard ratio;IR, incidence rate (per 1000 person-years); MACE, major adverse cardiac events; NA, not applicable; NODM, new onset diabetes mellitus; PSM, propensity score match.

## Supplementary Materials

The following are available online at http://www.mdpi.com/2077-0383/7/9/285/s1, Table S1: Propensity score model in the probability of statin prescription, Supplementary methods: Outcome measures, Identification of individual statins.

## References

[1] Schieppati A., Remuzzi G. (2005). Chronic renal diseases as a public health problem: Epidemiology, social, and economic implications. Kidney Int..

[2] Baigent C., Landray M.J., Reith C., Emberson J., Wheeler D.C., Tomson C., Wanner C., Krane V., Cass A., Craig J. (2011). The effects of lowering ldl cholesterol with simvastatin plus ezetimibe in patients with chronic kidney disease (study of heart and renal protection): A randomised placebo-controlled trial. Lancet.

[3] Jenkins M., Goldsmith D. (2012). Statins and kidney disease: Is the study of heart and renal protection at the cutting edge of evidence?. Curr. Opin. Cardiol..

[4] Palmer S.C., Craig J.C., Navaneethan S.D., Tonelli M., Pellegrini F., Strippoli G.F. (2012). Benefits and harms of statin therapy for persons with chronic kidney disease: A systematic review and meta-analysis. Ann. Int. Med..

[5] Trialists C.T. (2016). Impact of renal function on the effects of ldl cholesterol lowering with statin-based regimens: A meta-analysis of individual participant data from 28 randomised trials. Lancet Diabetes Endocrinol..

[6] Wanner C., Tonelli M. (2014). KDIGO Clinical Practice Guideline for Lipid Management in CKD: Summary of recommendation statements and clinical approach to the patient. Kidney Int..

[7] Sattar N., Preiss D., Murray H.M., Welsh P., Buckley B.M., de Craen A.J., Seshasai S.R.K., McMurray J.J., Freeman D.J., Jukema J.W. (2010). Statins and risk of incident diabetes: A collaborative meta-analysis of randomised statin trials. Lancet.

[8] Palmer S.C., Navaneethan S.D., Craig J.C., Johnson D.W., Perkovic V., Nigwekar S.U., Hegbrant J., Strippoli G.F. (2013). Hmg coa reductase inhibitors (statins) for dialysis patients. Cochrane Database Syst. Rev..

[9] Schurr J.W., Wu W., Smith-Hannah A., Smith C.J., Barrera R. (2016). Incidence of sepsis and mortality with prior exposure of hmg-coa reductase inhibitors in a surgical intensive care population. Shock.

[10] Wu V.C., Hu Y.H., Wu C.H., Kao C.C., Wang C.Y., Yang W.S., Lee H.H., Chang Y.S., Lin Y.H., Wang S.M. (2014). Administrative data on diagnosis and mineralocorticoid receptor antagonist prescription identified patients with primary aldosteronism in taiwan. J. Clin. Epidemiol..

[11] Wang W.J., Chao C.T., Huang Y.C., Wang C.Y., Chang C.H., Huang T.M., Lai C.F., Huang H.Y., Shiao C.C., Chu T.S. (2014). The impact of acute kidney injury with temporary dialysis on the risk of fracture. J. Bone Miner. Res..

[12] Cheng C.L., Kao Y.H., Lin S.J., Lee C.H., Lai M.L. (2011). Validation of the national health insurance research database with ischemic stroke cases in taiwan. Pharmacoepidemiol. Drug Saf..

[13] Charlson M.E., Pompei P., Ales K.L., MacKenzie C.R. (1987). A new method of classifying prognostic comorbidity in longitudinal studies: Development and validation. J. Chronic Dis..

[14] Bone R.C., Balk R.A., Cerra F.B., Dellinger R.P., Fein A.M., Knaus W.A., Schein R.M., Sibbald W.J. (1992). Definitions for sepsis and organ failure and guidelines for the use of innovative therapies in sepsis. Chest.

[15] Lin C.C., Lai M.S., Syu C.Y., Chang S.C., Tseng F.Y. (2005). Accuracy of diabetes diagnosis in health insurance claims data in taiwan. J. Formos. Med. Assoc..

[16] Lin Y.F., Lin S.L., Huang T.M., Yang S.Y., Lai T.S., Chen L., Wu V.C., Chu T.S., Wu K.D. (2018). New-Onset Diabetes After Acute Kidney Injury Requiring Dialysis. Diabetes Care.

[17] Wu V.C., Chueh S.C.J., Chen L., Chang C.H., Hu Y.H., Lin Y.H., Wu K.D., Yang W.S. (2017). Risk of new-onset diabetes mellitus in primary aldosteronism: A population study over 5 years. J. Hypertens..

[18] Wu V.C., Wu C.H., Huang T.M., Wang C.Y., Lai C.F., Shiao C.C., Chang C.H., Lin S.L., Chen Y.Y., Chen Y.M. (2014). Long-term risk of coronary events after aki. J. Am. Soc. Nephrol..

[19] WHO The Atc and DDD System. http://www.whocc.no/atc_ddd_index/.

[20] Tonelli M., Moye L., Sacks F.M., Kiberd B., Curhan G. (2003). Pravastatin for secondary prevention of cardiovascular events in persons with mild chronic renal insufficiency. Ann. Int. Med..

[21] LaRosa J.C., Grundy S.M., Waters D.D., Shear C., Barter P., Fruchart J.C., Gotto A.M., Greten H., Kastelein J.J., Shepherd J. (2005). Intensive lipid lowering with atorvastatin in patients with stable coronary disease. N. Engl. J. Med..

[22] Ou S.Y., Chu H., Chao P.W., Ou S.M., Lee Y.J., Kuo S.C., Li S.Y., Shih C.J., Chen Y.T. (2014). Effect of the use of low and high potency statins and sepsis outcomes. Intensive Care Med..

[23] Gupta R., Plantinga L.C., Fink N.E., Melamed M.L., Coresh J., Fox C.S., Levin N.W., Powe N.R. (2007). Statin use and hospitalization for sepsis in patients with chronic kidney disease. Jama.

[24] Dobesh P.P., Olsen K.M. (2014). Statins role in the prevention and treatment of sepsis. Pharmacol. Res..

[25] Patel J.M., Thickett D.R., Gao F., Sapey E. (2013). Statins for sepsis: Distinguishing signal from the noise when designing clinical trials. Am. J. Respir. Crit. Care Med..

[26] Koppe L., Pelletier C.C., Alix P.M., Kalbacher E., Fouque D., Soulage C.O., Guebre-Egziabher F. (2014). Insulin resistance in chronic kidney disease: New lessons from experimental models. Nephrol. Dial. Transplant..

[27] Kalantar-Zadeh K., Derose S.F., Nicholas S., Benner D., Sharma K., Kovesdy C.P. (2009). Burnt-out diabetes: Impact of chronic kidney disease progression on the natural course of diabetes mellitus. J. Ren. Nutr..

[28] Palmer S.C., Navaneethan S.D., Craig J.C., Johnson D.D., Perkovic V., Hegbrant J., Strippoli G.F. (2014). HMG CoA reductase inhibitors (statins) for people with chronic kidney disease not requiring dialysis. Sao Paulo Med. J..

[29] Hou W., Lv J., Perkovic V., Yang L., Zhao N., Jardine M.J., Cass A., Zhang H., Wang H. (2013). Effect of statin therapy on cardiovascular and renal outcomes in patients with chronic kidney disease: A systematic review and meta-analysis. Eur. Heart J..

[30] Chitalia N., Ross L., Krishnamoorthy M., Kapustin A., Shanahan C.M., Kaski J.C., Roy-Chaudhury P., Chemla E., Banerjee D. (2015). Neointimal hyperplasia and calcification in medium sized arteries in adult patients with chronic kidney disease. Semin. Dial..

[31] Wu P.C., Wu C.J., Lin C.J., Wu V.C. (2015). Long-term risk of upper gastrointestinal hemorrhage after advanced AKI. Clin. J. Am. Soc. Nephrol..

