# Supplementary material for: Effects of Statin Use in Advanced Chronic Kidney Disease Patients
Source: J Clin Med. 2018 Sep 17;7(9):285. doi: 10.3390/jcm7090285 (PMC6162375; doi:10.3390/jcm7090285)
Supplement: Supplementary file 1 [file jcm-07-00285-s001.pdf]

Supplementary files.

Supplementary methods

Outcome measures

The ICD-9 code of MI at hospitalization had high accuracy, as indicated by previous researches. [1,2] The records of CABG and angiography were reliable because they were constructed on the basis of TNHI propcedure codes that were tied to TNHI reimbursement system with routine auditing. The identification of patients with severe sepsis was similar to that used by Shen et al., [3,4] who selected all acute-care hospitalizations with ICD-9-CM codes for both a bacterial or fungal infection process and a diagnosis of acute organ dysfunction.

Identification of individual statins

Patients who had at least one prescription of Simvastatin (C10AA01), Lovastatin (C10AA02), Pravastatin (C10AA03), Fluvastatin (C10AA04), Atorvastatin (C10AA05), Rosuvastatin (C10AA07), and Pitavastatin (C10AA08) from the date of the first dose of ESA to 60 days before outcomes were identified in the TNHI prescription registry and defined as 'statin users'; the remaining patients were defined as 'statin nonusers'.

Table S1. Propensity score model in the probability of statin prescription

Variables	Odds Ratio (95% confidence interval)	*p* value
Age	0.974 (0.971,0.977)	<0.001
Men	0.517 (0.474,0.564)	<0.001
Hyperlipidemia	1.766 (1.537,2.028)	<0.001
Moderate or Severe liver disease	0.557 (0.444,0.691)	<0.001
Dipyridamole	1.201 (1.097,1.313)	<0.001
PPI	0.71 (0.596,0.842)	<0.001
Tumor	0.714 (0.578,0.874)	0.001
Hypertension	1.121 (1.018,1.235)	0.02
Diuretic	0.843 (0.769,0.924)	<0.001
Dementia	0.414 (0.184,0.803)	0.017
ACEI or ARB	1.103 (1.01 ,1.205)	0.029
Beta-Blocker	1.107 (1.008,1.214)	0.032
Ticlopidine	1.69 (1.033,2.686)	0.031

Abbreviations: ACEI, angiotensin-converting-enzyme inhibitors; ARB, Angiotensin II receptor blockers; PPI, proton-pump inhibitor.

References

1. Wu VC, Wu CH, Huang TM, et al. Long-term risk of coronary events after AKI. *J Am Soc Nephrol* 2014; **25**(3): 595-605.
2. Chu YT, Wu SC, Lee YC, Lai MS, Tam SC. Assessing measures of comorbidity using National Health Insurance Databases. *Taiwan J Public Health* 2010; **29**(3): 191-200.
3. Shen HN, Lu CL, Yang HH. Epidemiologic trend of severe sepsis in Taiwan from 1997 through 2006. *Chest* 2010; **138**(2): 298-304.
4. Lai TS, Wang CY, Pan SC, et al. Risk of developing severe sepsis after acute kidney injury: a population-based cohort study. *Critical care (London, England)* 2013; **17**(5): R231.
